# Occurrence of infective endocarditis following endoscopic variceal ligation therapy: A case report

**DOI:** 10.1097/MD.0000000000004482

**Published:** 2016-09-02

**Authors:** Xuan Zhang, Xiaoli Liu, Meifang Yang, Huihui Dong, Lichen Xv, Lanjuan Li

**Affiliations:** State Key Laboratory for Diagnosis and Treatment of Infectious Diseases, Collaborative Innovation Center for Diagnosis and Treatment of Infectious Diseases, The First Affiliated Hospital, College of Medicine, Zhejiang University, Hangzhou, China.

**Keywords:** case report, endoscopic variceal ligation, infective endocarditis, liver cirrhosis

## Abstract

**Background:**

Endoscopic variceal ligation (EVL) is the endoscopic treatment of acute esophageal variceal hemorrhage, however, prophylaxis antibiotic during EVL is controversial.

**Methods:**

We reported a 60-year-old man with diabetes, liver cirrhosis and hepatocellular carcinoma who received EVL for esophageal variceal haemorrhage.

**Results:**

On the second day after EVL, the patient developed fever and chills. A week after EVL, the blood cultures were viridans streptococcus positive, and echocardiogram showed a vegetation on the cardiac valve. The patient was therefore diagnosed with infective endocarditis (IE). The patient was cured after 7 weeks of intravenous piperacillin sulbactam sodium. No complications were observed during the 3-month follow-up after discharge.

**Conclusion:**

To our knowledge, this is the first documented case to report IE caused by viridans streptococcus after EVL. Therefore, whether prophylaxis antibiotic should be administered to cirrhotic patients receiving EVL is worth further research.

## 1 Introduction

In patients with liver cirrhosis, clinical complications are not uncommon, such as variceal hemorrhage, infection, ascites, and encephalopathy. Variceal hemorrhage is a dreaded complication, an important cause of morbidity and mortality. About one-third of the patients succumb to an initial bleeding episode due to failed noninvasive management.^[1]^ The survivors carry a 33% risk of reoccurrences of bleeding within 6 weeks, and this risk reaches up to 80% in the next 2 years.^[1]^ Endoscopic variceal ligation (EVL) is the endoscopic treatment of acute esophageal variceal hemorrhage, which is recommended by both American Association for the Study of Liver Diseases and the Baveno consensus.^[2,3]^

In this article, we report a rare case of infective endocarditis (IE) caused by viridans streptococcus after EVL for esophageal variceal hemorrhage in a cirrhotic patient without preceding symptoms of endocarditis. The case is presumed to be relevant to how to properly manage patients with acute esophageal variceal hemorrhage, and necessitates further study in potential complications following EVL administered to cirrhotic patients.

### 1.1 Case report

A 60-year-old man was admitted to our hospital in September 2014. The patient carried hepatitis B virus and was diagnosed with diabetes 3 years ago. He has been on insulin since then. Two years ago, the patient was confirmed to have hepatitis B type cirrhosis and hepatocellular carcinoma. Subsequently, radiofrequency ablation and transcatheter arterial chemoembolization were used to treat the cancer, and antiviral entecavir was also administered.

**Figure 1:**
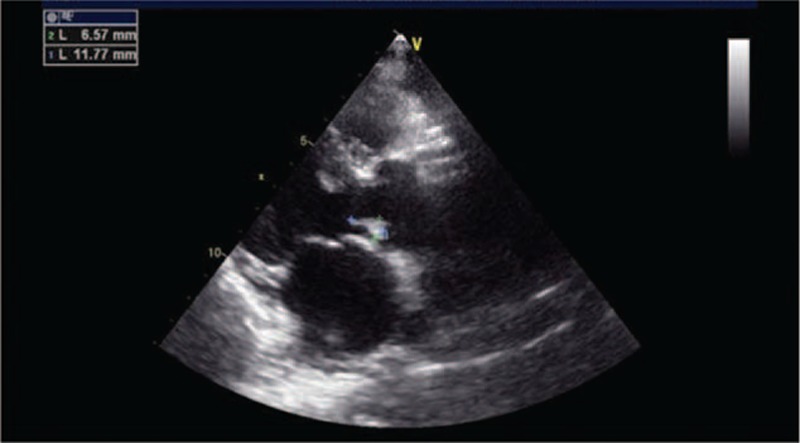
The echocardiogram showed a strong echo of 1.17∗0.66 cm at the valvula coronaria sinistra valvae aortae.

**Figure 2:**
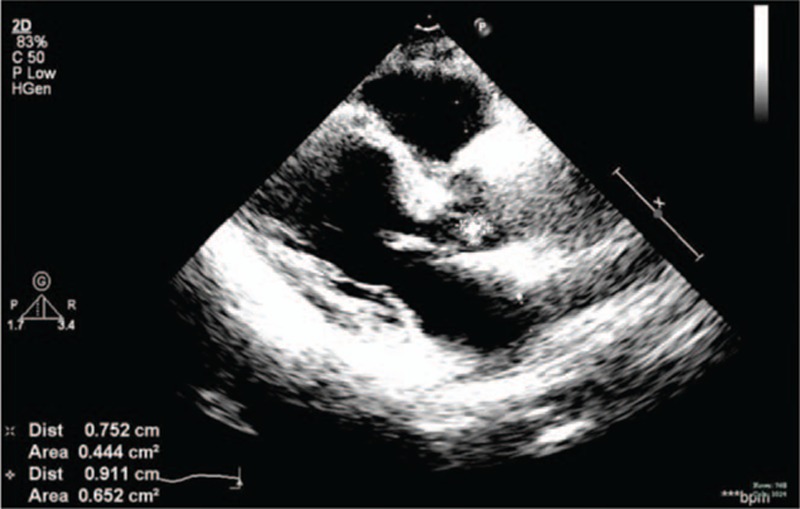
The echocardiogram presented a 0.91∗0.75 cm strong echo which was attached to the valvula coronaria sinistra valvae aortae.

**Figure 3:**
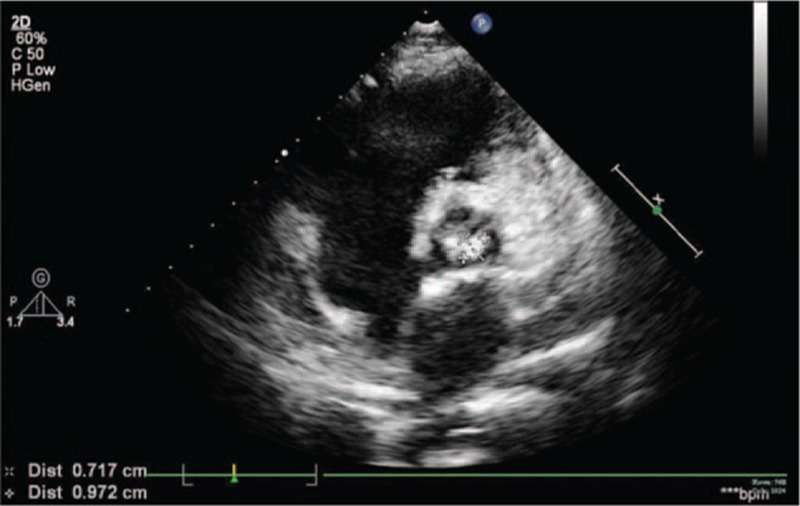
The echocardiogram showed a strong echo of 0.97∗0.72 cm at the valvula coronaria sinistra valvae aortae.

A month earlier, the patient experienced short episodes of upper gastrointestinal bleeding. After the bleeding had been controlled, the patient was admitted to our hospital for treatment of decompensated cirrhosis. He denied any symptoms of fever, toothache, pharyngalgia, chest congestion, or palpitation. Endoscopic esophageal band ligation had been done on September15, 2014, after relevant inspection. During the therapy, portal hypertensive gastropathy and duodenal ulcers were found. So we decided to give the patient a 24-hour fast, fluid infusion and proton pump inhibitor was also administered. In the evening of September 16, the patient developed fever and chills. The highest body temperature reached 38.9°C. The blood tests, include blood routine test, hypersensitive c-reactive protein, two blood cultures, were done immediately. Laboratory results were reported as follows (reference ranges are included in brackets): leukocyte count = 6.8 × 10E9/L (4–10 × 10E9/L), neutrophil 87.4%, hypersensitive C-reactive protein = 3.10 mg/L (0–8). Piperacillin sulbactam sodium for injection (2:1), at a dose of 4.5 g every 8 hours, was used at the same time. The body temperature returned to normal gradually. A week after the onset of fever, both the blood cultures were viridans streptococcus positive, which were sensitive to penicillin. So after the results of culture and antibiotic susceptibility testing were available, piperacillin sulbactam sodium was continued. At the same time, echocardiogram showed a strong echo of 1.17∗0.66 cm at the valvula coronaria sinistra valvae aortaetaa, and a mild aortic regurgitation (Fig. 1; Table 1). The conclusion was that the patient suffered from IE. After treatment for 7 weeks, echocardiogram also presented a 0.91∗0.75 cm strong echo that was attached to the valvula coronaria sinistra valvae aortae (Fig. 2), the body temperature was still normal. No other cardiovascular complications, like cardiac failure and embolism, were observed. For safety's sake, we had a consultation with the heart surgeon. The heart surgeon considered the patient suffered from liver cancer, which was a contraindication of extracorporeal circulation. So he suggested the patient should be managed conservatively. Subsequently, the patient was discharged from hospital after completing a 7-week course of piperacillin sulbactam sodium therapy. At the follow-up visit 3 months after the discharge, no symptoms or physical signs were observed, and the result of echocardiogram was similar to previous one (Fig. 3).

**Table 1:**
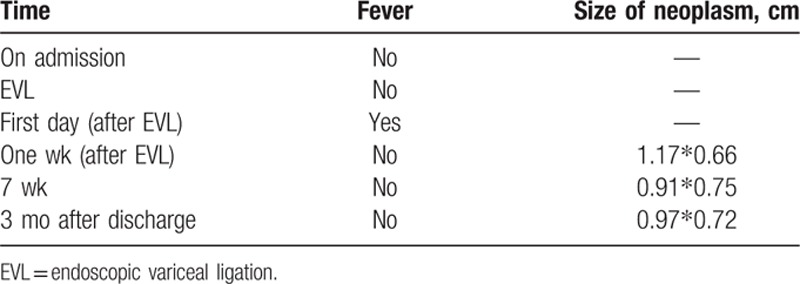
The change of symptom and echocardiogram.

## 2 Discussion

Despite recent advances in treatment, liver cirrhosis is one of the diseases that threaten the human life and health seriously. Liver cirrhosis reduces hepatic function and results in multiple complications induced by nodular regeneration and portal hypertension, including ascites, variceal bleeding, renal failure due to hepatorenal syndrome, hepatic encephalopathy, and spontaneous bacterial peritonitis.^[4]^ Research^[5]^ shows patients with cirrhosis are vulnerable to infection and aerobic Gram-negative bacilli are attributed more commonly.

In our case, the patient had many comorbidities, including diabetes, hepatitis B type cirrhosis, and liver cancer; antibiotic prophylaxis was not given after EVL. Subsequently, he suffered from bacteremia and IE caused by viridans streptococcus after EVL. We examined the complete clinical and ultrasound data following EVL; unfortunately, there was no echocardiogram before EVL. However, the patient denied history of heart diseases, symptoms of fever, toothache, pharyngalgia, chest congestion, or palpitation. Before EVL, the white blood count and hypersensitive C-reactive protein were both normal in the local hospital and our hospital. So the history and laboratory tests suggested that there was no IE before EVL.

IE is a deadly disease.^[6,7]^ Despite improvements in its management, IE remains associated with high mortality and severe complications. The 2015 European Society of Cardiology Guidelines^[8]^ recommend that antimicrobial treatment of native valve endocarditis should last 2 to 6 weeks. Meanwhile, the guidelines^[8]^ show that the risk of recurrence among survivors of IE varies between 2% and 6%; diabetes mellitus was a predictor of poor outcome in patients with IE. In our case, the patient was immunocompromised, diabetic with liver cirrhosis and live cancer. Moreover, the vegetation of cardiac valve was not shrinking obviously after anti-infective therapy. We considered that the patient had an increased rate of relapse. So the 7-week course of treatment was completed.

The 2015 European Society of Cardiology Guidelines^[8]^ recommend that antibiotic prophylaxis should be considered for patients at highest risk for IE, which included patients with any prosthetic valve, a previous episode of IE, and congenital heart diseases. Antibiotic prophylaxis is not recommended for gastroscopy, colonoscopy, cystoscopy, vaginal or caesarean delivery, or transesophageal echocardiography.^[8]^ Rerknimitr et al^[9]^ showed that prophylactic antibiotics may not be needed in nonbleeding gastric varices after endoscopic injection of cyanoacrylate. However, 2010 Asian Pacific Association for Study of the Liver^[10]^ recommended that third-generation cephalosporins should be given during EVL in acute variceal bleeding. A study^[11]^ showed that there was a significant risk of asymptomatic bacteremia and bacterial peritonitis after EVL. Meanwhile, Lin et al^[11]^ considered that the use of prophylactic antibiotics should be reserved for patients with Child's C class cirrhosis, a recent history of variceal bleeding, a history of bacterial peritonitis, or a comorbid immunosuppressive condition. Therefore, antibiotic prophylaxis during EVL has no clear consensus. In our opinion, it is necessary to use prophylactic antibiotics following EVL in some conditions.

Viridans streptococci are indigenous to the upper respiratory tract, the female genital tract, and the gastrointestinal tract, but are most prevalent in the oral cavity.^[12]^ There were reports^[12,13]^ that pyogenic meningitis occurred during the course of EVL. Liu et al^[12]^ reported a case of *Streptococcus sanguinis* meningitis. In our case, viridans streptococci may have survived the compromised host defense system to cause transient bacteremia after the invasive treatment under endoscope, followed by invasion of the cardiac valves.

## 3 Conclusion

To our knowledge, this is the first documented case of IE caused by viridans streptococcus after EVL. The cirrhotic patients are vulnerable to infection, which lead to high mortality and severe complications. Therefore, whether cirrhotic patients should receive prophylaxis antibiotic during EVL is worth further research.
